# Toward a Better Understanding of Shock Imprinting with Polymer Molds Using a Combination of Numerical Analysis and Experimental Research

**DOI:** 10.3390/ma15051727

**Published:** 2022-02-25

**Authors:** Kouki Hasegawa, Shigeru Tanaka, Ivan Bataev, Daisuke Inao, Matatoshi Nishi, Akihisa Kubota, Kazuyuki Hokamoto

**Affiliations:** 1 Graduate School of Science and Technology, Kumamoto University, 2-39-1 Kurokami, Chuo-ku, Kumamoto-shi 860-8555, Kumamoto, Japan; hasegawa@mech.kumamoto-u.ac.jp (K.H.); kubota@mech.kumamoto-u.ac.jp (A.K.); 2 Institute of Industrial Nanomaterials (IINa), Kumamoto University, 2-39-1 Kurokami, Chuo-ku, Kumamoto-shi 860-8555, Kumamoto, Japan; 3 Faculty of Mechanical Engineering and Technologies, Novosibirsk State Technical University, K. Marks 20, 630073 Novosibirsk, Russia; ivanbataev@ngs.ru; 4 Technical Division, Faculty of Engineering, Kumamoto University, 2-39-1 Kurokami, Chuo-ku, Kumamoto-shi 860-8555, Kumamoto, Japan; inao@tech.kumamoto-u.ac.jp; 5 Department of Mechanical and Intelligent Systems Engineering, National Institute of Technology (KOSEN), Kumamoto College, 2627 Hirayama-Shinmachi, Yatsushiro-shi 866-8501, Kumamoto, Japan; nishima@kumamoto-nct.ac.jp

**Keywords:** nanoimprinting, laser shock imprinting, high strain rate, polycarbonate, Autodyn

## Abstract

In the last decade, a new technique has been developed for the nanoimprinting of thin-metal foils using laser-induced shock waves. Recent studies have proposed replacing metal or silicone molds with inexpensive polymer molds for nanoimprinting. In addition, explosive-derived shock waves provide deeper imprinting than molds, greatly simplifying the application of this technology for mass production. In this study, we focused on explosive-derived shock waves, which persist longer than laser-induced shock waves. A numerical analysis and a set of simplified molding experiments were conducted to identify the cause of the deep imprint. Our numerical analysis has accurately simulated the pressure history and deformation behavior of the workpiece and the mold. Whereas a high pressure immediately deforms the polymer mold, a sustained pressure gradually increases the molding depth of the workpiece. Therefore, the duration of the pressure can be one of the conditions to control the impact imprint phenomenon.

## 1. Introduction

The nanostructures on the surfaces of metals exhibit unique electrical behavior depending upon their size and shape. For example, gold, silver, and copper nanostructures with nanogaps produce strong local electric fields owing to surface plasmons, which are useful for ultra-trace analysis in Raman spectroscopy owing to signal enhancement [1].

Moreover, such nanostructures exhibit a high optical absorption and are expected to be used in solar cells [2]. Surface nanostructures can be optically localized and can increase efficiency at certain wavelengths [3,4]. Metal nanostructures could be a new option in addition to semiconductors for use in solar cells. However, a major obstacle to the practical application of such structures is due to their lack of suitability for mass production.

Gao et al. developed a laser shock-imprinting (LSI) technology for the fast single-step fabrication of metallic nanostructures, using a Nd: YAG laser and a silicon nanomold prepared by electron beam lithography. The LSI process is rather simple: a laser-induced shock wave intensely presses a metal foil with a thickness of several micrometers against a Si nanomold, and the nanostructure is transferred onto the metal. Therefore, highly fine grooves of size 10 or 20 nm and pyramidal structures with sharp edges can be transferred onto the foil surface [5].

The fabrication of suitable molds is crucial for LSI technology. Si and Ni nanomolds, prepared by either nanoimprint lithography or electron beam lithography, are highly expensive. Moreover, a mold is essentially a consumable item in the LSI process [6]. With the development of soft lithography, research on the application of inexpensive polymer molds in the LSI process was introduced by compromising on imprinting accuracy for lower costs, which increased mass productivity. Later, metal arrays manufactured using polymer molds were observed to additionally possess excellent functionality. Specifically, Jin et al. [7] used a mold prepared by replicating the micrometer-order protrusions of bamboo leaves on an SU-8 polymer. The surface morphology of the bamboo leaves was subsequently transferred from the mold to the surface of an Al foil using LSI technology. This foil acquired the ability of water repellency and frictional power generation from raindrops rolling on its surface [7]. The fine trenches on an optical disk (CD, DVD, Blue-ray disk) that appear after peeling off the protective plate are also manufactured through soft lithography. Thus, optical disks are frequently used as cheap molds to test the LSI technique under laboratory conditions. Jin et al. [8] further studied LSI using a DVD mold. The trench geometry of this mold was a periodic structure with a period of 740 nm and peak of 130 nm. The constituent material of the mold was a metal deposited on a polycarbonate (PC) substrate. They showed that an Al foil imprinted with the trench-shaped DVD mold generated photovoltaic power, owing to surface plasmon resonance [8].

**Figure 1:**
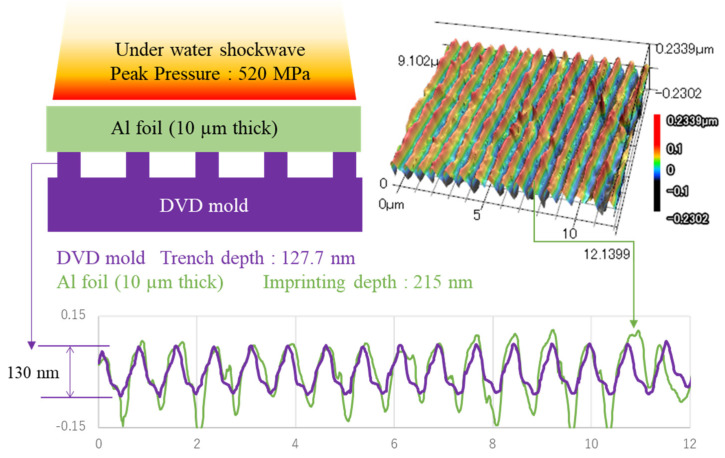
Imprint experiment: an Al foil was compressed into a DVD mold by an underwater shock wave derived from an explosive, yielding a deeper imprinting shape than the DVD mold.

It is difficult to process a large area with LSI because of the small laser spot diameter. The application of underwater shock waves derived from explosives has emerged as a novel approach toward shock-imprinting technology. The explosive-derived shock waves achieve large-area imprinting in a single shot; imprints of the micrometer order, covering an area of 100 mm × 100 mm, using musing plant leaves directly as molds have already been demonstrated [9]. Furthermore, shock-imprinting studies using explosives and DVD molds have been conducted for comparison with LSI; a high molding imprinting accuracy (117 nm imprint on 130 nm mold) has been achieved with a single shot [10]. Additionally, one-dimensional nanoimprinting through linear explosives has lifted the processing size restrictions [11]. We performed an imprinting experiment in which an Al foil was compressed into a DVD mold by an underwater shock wave derived from an explosive. A schematic including the results is shown in Figure 1 (the detailed experimental conditions were reported by Tanaka et al. [10]). The Al foil exhibits a deeper imprinting than the DVD mold. The factors causing this peculiar imprinting phenomenon have not been explored. However, pressure duration could be one of the factors. The duration of a laser-induced shock wave is equal to approximately three times that of a laser pulse duration [12], which is marginally shorter than 100 ns [13]. On the other hand, the duration of explosive-derived shock waves is considerably longer than that of laser shocks.

In submicron-order processing, the surface roughness of the starting material affects the final imprinting shape of the sample [14], and the local deformation and fracture of the workpiece are highly dependent on its grain size [15]. These are the factors that complicate the shock-imprinting phenomenon in submicron-order processing.

This study explores the factors that contribute to the appearance of deep imprinting. We performed submillimeter-order molding experiments on Al workpieces using explosive-derived underwater shock waves and PC molds to prepare specimens for comparison with numerical analysis and avoid complexities due to the heterogeneity of the starting material. The explosive, pressure transmitting medium (water), Al workpiece, and PC mold were simulated numerically using the existing equations of state and constitutive laws. Additionally, the dynamical behavior of the metal workpiece and mold during the shock compression process were analyzed.

## 2. Experimental Section

**Figure 2:**
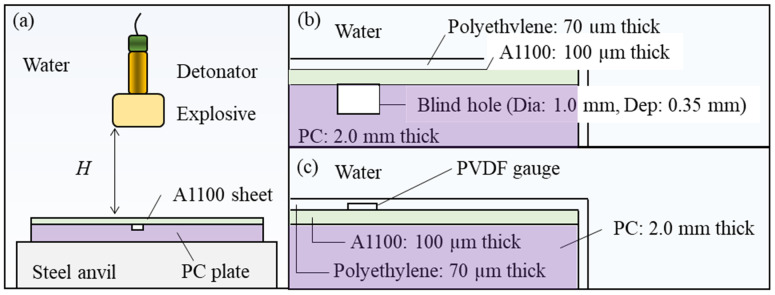
Schematic illustration of the shock-imprinting and pressure measurement experiments using a PC mold. (**a**) The layout of an explosive and sample in water. (**b**) Details of the placement of the PC mold and Al workpiece. (**c**) Details of the PVDF gauge placement.

The purpose of our experiment was to prepare samples to be compared with the numerical analysis results and measure the pressure acting on the samples. The outline of the experimental apparatus is shown in Figure 2. Figure 2a shows the arrangement of the explosives and samples. In this study, explosives were used to produce shock waves. Three grams of SEP explosive manufactured by Kayaku Japan Co., Ltd. (Tokyo, Japan) was molded into the shape of a cylinder with a diameter of 18 mm and placed in water. The main component of SEP is pentaerythritol tetranitrate, and its detonation velocity and density are 6970 m/s and 1310 kg/m^3^, respectively. The SEP explosive was detonated by the No. 6 electric detonator manufactured by Kayak Japan Co., Ltd. The area of action of the underwater shock wave generated by this explosion was overwhelmingly larger than the molding area of the sample; therefore, the pressure applied to the workpiece and mold was considered uniform. The metal sample used in the experiment was an Al alloy (A1100) manufactured by Niraco Co., Ltd. (Tokyo, Japan), and its thickness was 100 µm. The mold was a PC plate provided by Takiron Co., Ltd. (Osaka, Japan), and its thickness was 2.0 mm. The PC plate contained a blind hole on one side with a diameter of 1.0 mm and a depth of 0.35 mm. The mold and the Al workpiece were placed in a vacuum polyethylene bag with a thickness of 70 µm and evacuated to an internal pressure of 0.01 MPa, as shown in Figure 2b. They were placed on a steel anvil at a distance *H* from the explosive. A set of imprinting experiments were conducted with distance *H* in the range of 20 to 100 mm. After imprinting, the sample was studied with JCM-5700, a scanning electron microscope (SEM) manufactured by JEOL Ltd. (Tokyo, Japan).

Another set of experiments were performed to accurately measure the shock wave pressure. The pressure history was used to ensure the accuracy of the subsequent numerical simulations. When an explosive charge detonates in water, an underwater shock wave is generated. Water is a suitable medium for transmitting the pressure and protecting the sample from the heat of the explosion. The shock pressure received by the Al workpiece depends on the distance *H*. The pressure history applied to the Al workpiece was evaluated using a polyvinylidene difluoride (PVDF) gauge, as was described in our previous study [16]. The PVDF gauge, PVF2-11-125-EK (Dynasen, Inc., Goleta, CA, United States), was glued to the surface of the Al workpiece, layered on the PC plate. In addition, 70 µm-thick polyethylene was glued to it (Figure 2b). The PC plate and Al workpiece were the same as in the imprinting experiment. After the underwater shock waves acted vertically on the PVDF gauge, the pressure signal was displayed on the oscilloscope (DPO7254C, Tektronix, Inc., Beaverton, OR, USA) via the integrator.

## 3. Numerical Simulation

For the numerical analysis, we used ANSYS Autodyn 19.2, a shock analysis code capable of complex modeling and simulating explosions. The code uses the Rankine–Hugoniot equation and other equations to calculate the equation of state (EOS), which is coupled with the conservation equations for mass, momentum, and energy to calculate the leap condition specific to impact phenomenon. Autodyn can use Lagrangian, Eulerian, arbitrary Lagrangian–Eulerian, smoothed-particle hydrodynamics, and other solvers. Additionally, the interactions between these solvers can also be considered, which is widely used in impact analysis.

In analyses involving explosives, if the detonation process of the explosive is calculated using a rough computational mesh, the detonation pressure of the explosive will be underestimated, and the calculation results will not be meaningful. Moreover, if the explosives are relatively small in a wide analysis range, it will be difficult to obtain a fine computational mesh for the explosives. To solve this problem, Autodyn uses a remap function that calculates a fine mesh only for the explosive part and maps the result to a wide calculation area. In the remap function, this is achieved by embedding the physical quantities, such as compressibility, specific internal energy, and velocity, calculated in the fine computational domain into the wide computational domain. If the size of the explosive is relatively smaller than the calculation area, or if the distance to the explosive is relatively larger than the calculation area, etc., there will be a difference in size of the calculation area when modeling; the remap function will limit the calculation area so that the calculations are accurate. In this study, underwater shock waves were derived from explosives and water placed dozens of millimeters apart, while the imprinting region consisted of a relatively small 0.1 mm-thick Al workpiece and mold; therefore, the generated underwater shock waves were remapped to a small imprinting region to accurately simulate the experimental model.

**Figure 3:**
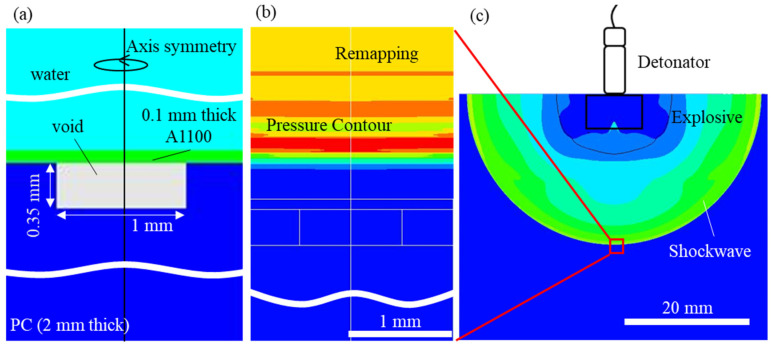
Numerical-simulation model using 2D Lagrangian and Eulerian solvers. (**a**) Dimensions and shapes of the PC mold, Al workpiece, and water. (**b**) Setting of the initial conditions. (**c**) Underwater shock-wave model consisting of the explosive and water.

In the numerical model, the explosives and water were placed several tens of millimeters apart, the thickness of the Al workpiece was set as 0.1 mm, and an axis-objective 2D Lagrangian and Eulerian solver interaction was used with respect to the y-axis. The model diagram is shown in Figure 3a. The PC mold was set at a diameter of 1 mm with a hole depth of 0.35 mm. A 4 mm × 5 mm area of water was modeled around the Lagrangian model consisting of the Al plate and the PC mold using a Eulerian solver. In this analysis, everything was divided into 0.01 mm quadrilateral meshes.

The pressure acting on the top surface of the Al plate varies depending on the material and deformation superimposed on the Al. An accurate model of the underwater shock wave is necessary to accurately simulate the deformation process. Therefore, in this computational model, water was composed of Eulerian elements, and Al and PC were composed of Lagrangian elements. This model used the interaction between the Eulerian and Lagrangian elements to perform the calculations. The shock-wave waveforms at certain distances from the explosives (*H* = 20, 40, and 60 mm) were calculated using a computational model consisting of explosives and water. Figure 3c shows the simulated underwater shock wave. These shock-wave waveforms were extracted from physical quantities, such as compressibility, specific internal energy, and velocity. The calculated results were embedded in a small computational domain consisting of Al and PC to simulate the condition just before the underwater shock wave reached the Al under the constraint that *H* was between 20 mm and 60 mm (Figure 3b). 

An underwater shock wave consisting of explosives and water was modeled on a Eulerian mesh of a two-dimensional axial target system. Two EOS were used in this model.

(1) $$p=A\left(1-\frac{\omega }{R_{1}V}\right)e^{-R_{1}V}+B\left(1-\frac{\omega }{R_{2}V}\right)e^{-R_{2}V}+\frac{\omega e}{V},$$

Equation (1) is based on the Jones–Wilkins–Lee (JWL) EOS for explosives, where *p* is the pressure; *V* is the ratio of the initial density of the explosive to the density of the explosive gas products; *e* is the internal energy; and *A*, *B*, *R*_1_, *R*_2_, and *ω* are the empirical coefficients.

The Mie–Gruneisen-type EOS was used for the water model; the details of this EOS are explained in the remapped analytical model.

The model consisting of explosives and water was divided into 0.05 mm quadrilateral meshes. Calculations were performed for distances of *H* = 20, 40, and 60 mm from the explosives and then remapped to the model consisting of the Al workpiece and PC mold.

SEP was used as the explosive, and its parameters for JWL EOS are listed in Table 1.

The material boundaries were assumed to be continuous, and the movement was restricted to the longitudinal direction only. A1100, PC, and water were used in this model. These materials were modeled using the Mie–Gruneisen EOS based on Hugoniot data (Equations (2)–(5)).

(2) $$p=p_{H}+\Gamma_{\rho }\left(e-e_{H}\right),$$

(3) $$\Gamma_{\rho }=\Gamma_{0}\rho_{0}=const,$$

(4) $$p_{H}=\frac{\rho_{0}c_{0}^{2}\mu \left(1+\mu \right)}{{\left(1-\left(s-1\right)\mu \right)}^{2}},$$

(5) $$e_{H}=\frac{1}{2}\frac{\rho_{H}}{\rho_{0}}\left(\frac{\mu }{1+\mu }\right).$$

Here, *p* is the pressure, *P_H_* is the Hugoniot pressure, *Γ* is the Gruneisen gamma, *ρ* is the density, *e* is the internal energy, $e_{H}$ is the Hugoniot internal energy, *Γ_ρ_* is the Gruneisen gamma of the reference state, $\rho_{0}$ is the initial density, $c_{0}$ is the bulk velocity of the sound, *μ* is the compression, and *s* is the linear Hugoniot gradient coefficient.

The parameters of the Mie–Gruneisen EOS for A1100 and PC are shown in Table 2.

An accurate characterization of the mechanical properties of materials subjected to high-strain-rate deformation is very complex because of the simultaneous effects of several phenomena, such as workpiece hardening, strain-rate hardening, and thermal softening. In this case, the yield stresses of A1100 and PC were estimated using the Johnson–Cook elasto-viscoplastic material model [20]. The equivalent von Mises yield stress (*σ_y_*) is defined as follows:

(6) $$\sigma_{y}=\left(A+B\varepsilon_{p}^{n}\right)\left(1+Cln\dot{\varepsilon_{p}^{*}}\right)\left(1-T^{*m}\right),$$

where *A*, *B*, *n*, *C*, and *m* are the material parameters; *ε_p_* is the equivalent plastic strain; $\dot{\varepsilon_{p}^{*}}=\dot{\varepsilon }/\dot{\varepsilon_{ref}}$ is the dimensionless strain rate; and T* is the dimensionless temperature. The dimensionless temperature is defined as $\left\{T^{*}=\left(T-T_{room}\right)⁄\left(T_{m}-T_{room}\right)\right\}$. Additionally, $\dot{\varepsilon }$ is the strain rate, $\dot{\varepsilon_{ref}}$ is the reference strain rate, *T* is the current temperature, *T_m_* is the melting temperature of the alloy, and $T_{room}$ is the room temperature.

As the punching phenomenon is closely related to material failure, the Johnson–Cook fracture model [21] was used in the numerical analysis to analyze the behavior of A1100 and PC under high strain conditions. In this model, the equivalent strain to fracture $\varepsilon_{f}$ is defined as follows:

(7) $$\varepsilon_{f}=\left(D_{1}+D_{2}e^{D_{3}\sigma^{*}}\right)\left(1+D_{4}ln\dot{\varepsilon_{p}^{*}}\right)\left(1+D_{5}T^{*}\right),$$

where *D*_1_ to *D*_5_ are the material parameters, $\sigma^{*}=\sigma_{m}⁄\sigma_{eq}$ is the stress triaxial ratio, *σ_m_* is the mean stress, and *σ_eq_* is the equivalent von Mises stress. Depending on the equivalent fracture strain, the damage parameters can be calculated as follows:

(8) $$D=\Sigma \frac{\Delta \varepsilon }{\varepsilon_{f}},$$

Here, *D* is the damage parameter, which is assumed to be equal to one when the material is destroyed and intact until then, and *Δε* is the plastic strain.

The parameters of the Johnson–Cook constitutive law and fracture law for A1100 and PC used in this analysis are listed in Table 3.

## 4. Results and Discussion

### 4.1. Experimental Results

**Figure 4:**
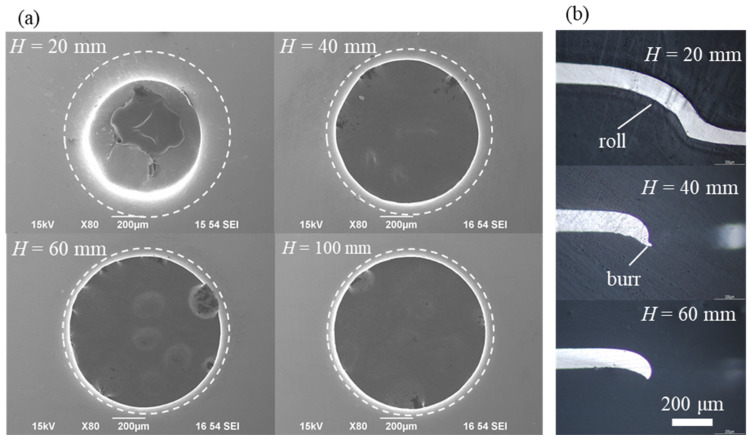
Final shape of an Al workpiece under varying pressure conditions. (**a**) Surface of the Al workpiece at pressure-loading side. (**b**) Cross-section of the workpiece.

SEM images of the Al samples after experiment are shown in Figure 4. In most of the cases, a through-hole was formed in the samples. Surprisingly, complete punching was not observed, specifically for the case of *H* = 20 mm, i.e., for the experiment with the highest pressure applied to the foil. This effect was later clearly simulated using numerical analysis, and it is discussed in the next subsection. The diameter of the dashed circle, depicted in Figure 4a, is 1.0 mm. The diameter of the punched hole for *H* = 40 mm (0.85 mm) is slightly smaller than that for *H* = 60 mm (0.92 mm). The punching diameters for *H* = 80 mm and *H* = 100 mm are 0.91 mm and 0.93 mm, respectively (i.e., almost the same values as that at *H* = 60 mm). The cross-sections of the holes formed under the conditions with *H* in the range from 20 to 60 mm are shown in Figure 4b. The cross-section of the sample produced at *H* = 20 mm had a longer roll section than the other samples, implying that the mold deformed synchronously with the workpiece. In the cross-section of samples with *H* = 40 mm and *H* = 60 mm, no significant difference was observed. However, burrs caused by ductile fracture were observed in the cross-section for *H* = 40 mm. In previous studies that used metal punching molds and Al or Cu workpieces, punching holes were formed by the ductile fracture of the workpiece [13,23], and the fracture process was similar to that described in the current study. The pressure measurement results are described in detail in the next section for comparison with the results of the numerical simulations.

### 4.2. Numerical Simulation Results

**Figure 5:**
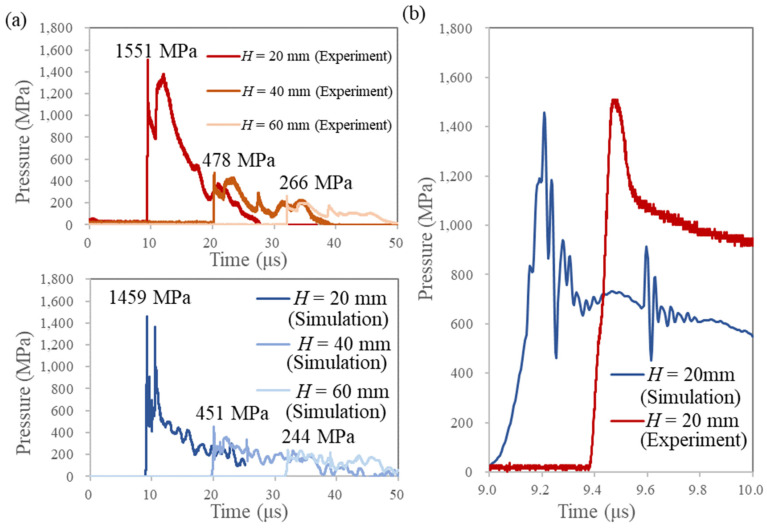
Comparison of the pressure histories received from experimental and numerical simulations. (**a**) Comparison of the peak pressure values and shapes at different values of *H*. (**b**) Comparison of the pressure rise process for *H* = 20 mm.

The pressure profile shown in Figure 5 represents the reflected pressure of the underwater shock wave on the surface of the Al workpiece, which was obtained by experimental and numerical analyses. The installation of a PVDF gauge on a rigid body is the supplier’s recommended method of application. The typical pressure profile of the shock wave obtained in this case is a triangle, representing a sharp rise and decay in pressure, with only one peak [16]. In this study, a characteristic profile with two peaks was obtained, which can be attributed to the deformation of the PC plate. The rapidly increasing pressure indicated the arrival of the underwater shock wave and formation of the first peak; the PC plate behind the Al work was compressed by the high pressure and then the pressure decreased rapidly. When the compressive deformation subsided, the pressure increased again and formed the second peak. Both the experimental and numerical results showed a characteristic pressure profile with two peaks. Furthermore, the pressure values of the first peak are in good agreement, indicating that the numerical simulation is accurate (Figure 5a).

When a thin-metal workpiece is subjected to a shock wave, as in this study, it starts to deform immediately. Therefore, the first pressure peak and the rise time (time required to reach the first peak pressure from 0 Pa) are crucial factors for the deformation analysis of the workpiece. The effect of the pressure rise time on the deformation behavior in the numerical analysis is discussed in the next paragraph after the following general observations regarding the rise time obtained in the experiment and the simulation. For a more accurate comparison of the experimental and simulation results, the pressure profiles for the case of *H* = 20 mm are shown in Figure 5b. The pressure rise times were 130 ns and 180 ns in the experiment and numerical simulation, respectively. This duration is short, but not infinitely small. However, shock waves are considered as pressure-jump phenomena, and the physical quantity shows discontinuous changes. In other words, the pressure rise time should be equal to zero in the actual phenomenon. The finite value of rise time in the experiment was due to the finite thickness of the PVDF gauge. The active surface of the PVDF gauge was 0.028 mm thick; the smaller this thickness is, the steeper the pressure rise will be in the measurement. Additionally, the non-zero rise time in the numerical simulation was due to other factors. The use of the remap function in the numerical simulation caused the simulated rise time to be slightly longer than that in the experiment. The pressure rise time in the numerical analysis will be closer to the actual phenomenon if a finer mesh size is used in the calculations. However, this issue was beyond the scope of this study because it requires a specialized high-performance computer.

**Figure 6:**
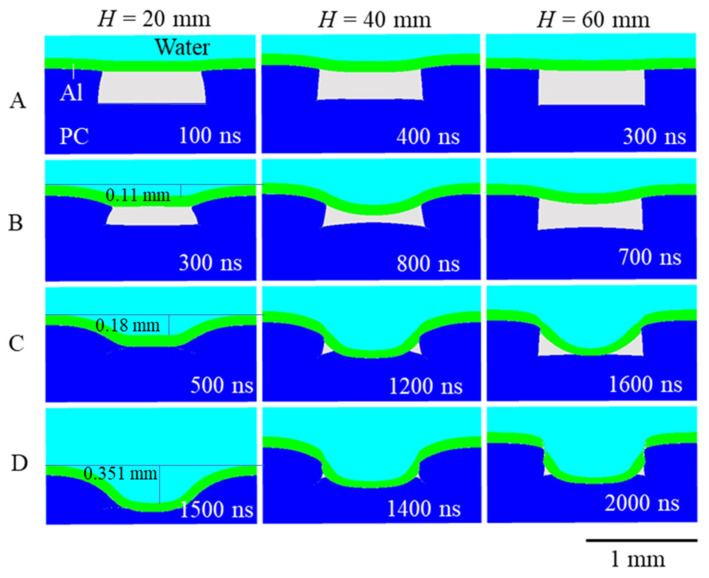
Deformation of the workpiece and mold due to the underwater shock wave.

The deformation process of the Al workpiece and the PC mold simulated by the numerical analysis for *H* in the range of 20–60 mm is shown in Figure 6. The time in the figure is the elapsed time, with 0 ns being the time when the Al workpiece was subjected to the shock pressure. The higher the pressure of the shock wave was, the earlier the deformation of Al and PC started (line A in Figure 6). 

In the high-pressure condition (*H* = 20 mm), the PC mold did not maintain its shape and deformed significantly, whereas, in the low-pressure condition (*H* = 60 mm), it maintained its shape (line B in Figure 6). For the *H* = 40 and 60 mm cases, a decrease in the thickness of the Al workpiece near the edge of the mold was simulated, while in the case of *H* = 20 mm, no such local decrease occurred, owing to the large deformation of the mold (line C in Figure 6). The reason that the Al workpiece was not punched in the high-pressure condition (*H* = 20 mm) was clearly because of the deformation of the mold; the edge of the mold was squeezed and did not cut the foil. In the simulation for *H* = 40 mm, the Al workpiece was not punched; however, a noticeable localization of deformation was observed near the edge of the mold. For the *H* = 60 mm case, the Al workpiece was punched (line D in Figure 6). Note that the deformation continued after the workpiece reached the bottom of the mold in the case of *H* = 20 mm. This was due to the continuous compression of the Al workpiece by the underwater shock wave from the explosive, which maintained a high pressure for a long period of time in the order of microseconds. The nanoimprinting studies using explosive-derived shock waves and DVD molds have yielded molding accuracy values that are far superior to the LSI studies, providing deeper relief on the sample surface [10,11]. From this simulation, it may follow that a higher molding accuracy was achieved, owing to the long pressure duration and the flexibility of the polymer mold, which deformed significantly during the process and did not cut the foil. 

Under the condition of *H* = 40 mm in the numerical analysis, the workpiece did not fracture. This was because the PC mold deformed slightly more than in the experiment. The pressure rise time (180 ns) observed in Figure 5b possibly deviated the deformation behavior in the numerical analysis from the actual phenomenon. This should be significant for simulating micrometer-order imprinting. In the present study, as the simulation was conducted for a relatively large imprinting geometry, the amount of deformation during the rise time was small even under high-pressure conditions, and the effect of the rising time was neglectable.

**Figure 7:**
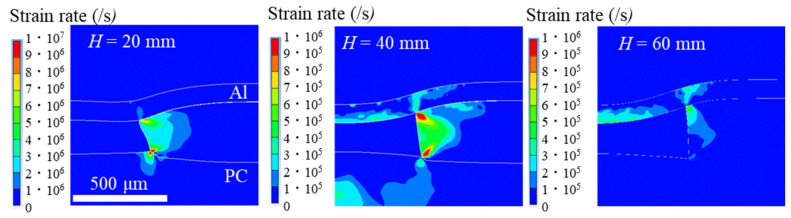
High strain-rate regions during imprinting.

The strain rates of the workpiece and mold at specific times for each condition are shown in Figure 7. Specific time refers to the time when the maximum strain rate was output. The contours in Figure 7 show 10^7^ and 10^6^/s for *H* = 20 mm and other conditions, respectively. This indicates the strain-rate region, where the dislocation avalanche can occur in the work piece. In the LSI studies using Si nanomolds, the workpiece deformation is constrained to the edges of the mold at strain rates >10^5^/s, resulting in a high dislocation density and dislocation avalanches in the workpiece (large deformation) [5,11,24]. On the other hand, when a polymer mold was used, the mold deformed significantly under pressure conditions in the order of gigapascals (Figure 7) (*H* = 20 mm). Therefore, it became difficult for the transition to concentrate on the work piece at the edge of the mold, and the dislocation avalanche did not occur. Even in the sub-micrometer-order shock-imprinting phenomenon, polymer molds could not maintain their shape under high-pressure conditions. The imprinting mechanism using the polymer mold was essentially different from that using the rigid silicon mold.

In the shock materials processing including LSI and the current study, the mold and workpiece deformed at a high strain rate, and the strength of many metallic materials, including Al, depended on the strain rate. PC also possesses a significant strain-rate hardening effect [25]. For the Johnson–Cook constitutive law, which considers the change in material strength with the strain rate, the stress–strain curve of the material shifts upwards as the strain rate increases. Therefore, the higher the pressure, the higher the yield stress of the workpiece. However, even if a strain rate of 10^7^/s is achieved during the imprinting, Equation (6) indicates that the yield strength would only increase by a factor of 1.26 for Al and 1.84 for PC. Therefore, the increase in strength of the PC mold due to the increase in the strain rate does not significantly affect the deformation process in shock imprinting (at least when the pressure is of the order of 1 GPa (*H* = 20 mm)).

## 5. Conclusions

In this study, we conducted molding experiments of Al workpieces with relatively large sizes and pressure-measuring experiments using explosive-derived underwater shock waves and compared the experimental results with the numerical simulation results. This study revealed that the duration of pressure is one of the conditions to control the shock-imprinting phenomenon. Our pressure-measuring experiment using PC as a substrate for a PVDF pressure gauge was successful in measuring a pressure profile, which reflected the compressive deformation of the substrate. The pressure profile obtained by numerical analysis simulated the experimental pressure with two pressure peaks, which strongly supported the experimental results. Furthermore, both the experiment and simulation showed good agreement in the case of peak pressure. A numerical model composed of explosives and water was integrated into the deformation analysis model to simulate the deformation process of the Al workpiece and PC mold. We found that the effect of the change in the mold shape owing to the applied shock pressure was the dominant factor in determining whether punching was achieved according to the pressure conditions. Under pressure conditions in the order of gigapascals, the effect of the strain rate on the increase in PC strength was negligible; explosive-derived shock waves that sustained for the order of microseconds, coupled with the flexibility of the polymer mold, increased the molding depth. The equation of state, constitutive laws, and parameters applied to the material model in the study’s numerical analysis accurately simulated the dynamic deformation phenomena. The limitations of this study include the inability to justify how to scale various orders of magnitude and how to interpret results obtained at the millimeter scale to the nanometer scale. Previous LSI research had focused on controlling the pressure generated, assuming that a stronger pressure will result in a faster drive of the workpiece, and thus an improved imprinting accuracy. The results of this research will add a new consideration (pressure duration) to shock-imprinting research. Relatively thick metal workpieces were not the target of shock imprinting in previous studies. Controlling the pressure and its duration may not only relax the workpiece thickness limitation, but also improve the imprinting accuracy. Attempts to use polymer molds for shock-imprinting research were introduced under the premise of compromising the molding accuracy for cost. However, controlling the duration of pressure will allow for both mass production and a high imprinting accuracy.

## Figures and Tables

**Table 1:** Jones–Wilkins–Lee (JWL) equation of state (EOS) parameters for the SEP explosive [16].

Parameter	Value
Reference density (g/cm^3^)	1.31
*A* (kPa)	3.65 × 10^8^
*B* (kPa)	2.31 × 10^6^
*R*_1_	4.3
*R*_2_	1.0
Ω	0.28
C-J energy/unit volume *e* (kJ/m^3^)	3.761 × 10^6^
C-J detonation velocity (m/s)	6.97 × 10^3^
C-J Pressure *P_CJ_* (kPa)	1.59 × 10^7^

**Table 2:** Mie–Gruneisen EOS parameters for A1100 [17], PC [18] and water [19].

	A1100	PC	Water
Reference density (kg/m^3^)	2.707	1.197	1.00
Gruneisen gamma	1.970	0.61	0.28
$c_{0}$ (m/s)	5386	1933	1483
*s*	1.339	2.6050	1.75
Reference temperature (K)	293	300	-
Specific heat (J/kg·K)	884	-	-

**Table 3:** Johnson–Cook strength and failure models parameters for A1100 [8,22] and PC [8].

	A1100	PC
*A* (MPa)	140	75.8
*B* (MPa)	157	68.9
*C*	0.016	0.052
*n*	0.167	1
*m*	1.7	1.85
*D*_1_	0.071	-
*D*_2_	1.248	-
*D*_3_	−1.142	-
*D*_4_	0.0097	-
*D*_5_	0.0	-

## Data Availability

Data sharing not applicable.
